# Predictors and moderators of outcome of ICBT for loneliness with guidance or automated messages - A secondary analysis of a randomized controlled trial

**DOI:** 10.1016/j.invent.2023.100701

**Published:** 2023-12-18

**Authors:** Noëmi Seewer, Andrej Skoko, Anton Käll, Gerhard Andersson, Thomas Berger, Tobias Krieger

**Affiliations:** a Department of Clinical Psychology and Psychotherapy, University of Bern, Bern, Switzerland; b Department of Behavioral Sciences and Learning, Department of Biomedical and Clinical Sciences, Linköping University, Linköping, Sweden; c Center for Social and Affective Neurosciences, Linköping University, Linköping, Sweden; d Department of Clinical Neuroscience, Karolinska Institute, Stockholm, Sweden

**Keywords:** Loneliness, ICBT, Guidance, Automated messages, Predictor, Moderator

## Abstract

Internet-based cognitive behavioral therapy (ICBT) is promising in alleviating loneliness in adults. Identifying individuals who benefit from ICBT for loneliness is pivotal to offering this intervention in a more targeted way and improving the intervention for those who do not benefit. This secondary analysis of a randomized controlled trial (RCT) aimed to identify predictors and moderators of outcome of an ICBT with guidance or automated messages for loneliness. In the RCT, 243 participants suffering from loneliness were randomly assigned to an ICBT with guidance (*n* = 98), automated messages (*n* = 97), or a waitlist-control condition (*n* = 48). In total, 180 participants completed the post-assessment (i.e., 10 weeks post-randomization). Outcomes were treatment outcome assessed with the UCLA-9 Loneliness Scale at post-assessment and treatment response, i.e., reliable improvement on the UCLA-9 from pre- to post. The relationship between a wide range of patient characteristics (grouped into socio-demographic, clinical, loneliness-specific, and treatment-related variables) and outcome was analyzed using multiple linear and logistic regressions. Feeling less burdened by loneliness resulted in higher odds of reliable improvement in guided ICBT compared to the waitlist-control condition. No treatment outcome or response moderators were identified for ICBT with automated messages compared to the waitlist-control group. Across active intervention groups, loneliness at baseline, age and fit between the tasks and goals of the intervention and participants' need predicted treatment outcome. Predictors of treatment response for ICBT with guidance and automated messages were not identified, and no variables differentially predicted the effects of ICBT with guidance or automated messages on the outcomes. In conclusion, individuals less burdened by their feelings of loneliness benefited more from guided ICBT. Lower baseline loneliness scores, younger age, and a better match between tasks and goals of the intervention and participants' needs also predicted a more favorable treatment outcome for both ICBT with guidance and automated messages.

## Highlights

• First study to investigate predictors and moderators of treatment outcome and response of ICBT for loneliness with or without guidance.
• Higher odds ratios of reliable improvement in those feeling less burdened by loneliness in guided ICBT vs.waitlist.
• No treatment outcome or response moderators identified for ICBT with automated messages vs. waitlist.
• Lower baseline loneliness, younger age, and matching intervention tasks and goals with user needs predicted better outcome.
• No variables differentially predicted the treatment outcome or response of an ICBT with guidance or automated messages.

## 1 Introduction

### 1.1 Introduction

Loneliness is an aversive subjective experience resulting from a discrepancy between actual and desired social relationships (Peplau and Perlman, 1982). In Western European countries, prevalence rates of adults suffering from loneliness span from 4.9 % (18–29 years), 5.1 % (30–59 years) to 8.7 % in the oldest adults (≥ 60 years) (Surkalim et al., 2022). Furthermore, loneliness is linked to various adverse mental health outcomes, including depression, social anxiety, suicidality, lower general mental health, and overall wellbeing (see Park et al., 2020, for a review). Thus, loneliness is recognized as an important public health issue (Holt-Lunstad, 2022).

### 1.2 Interventions for loneliness

Various interventions to reduce loneliness have been developed and evaluated. Interventions targeting maladaptive social cognitions have been among the most effective in reducing loneliness (Masi et al., 2011). Maladaptive social cognitions in lonely individuals include, for example, expecting others to reject them or evaluating themselves and others negatively (Spithoven et al., 2017). Changing cognitions is a crucial aim in cognitive behavioral therapy (CBT), and CBT has increasingly been delivered in the form of Internet-based cognitive behavioral therapy (ICBT) during the past decades for various mental health conditions (Andersson, 2018). ICBTs are low-threshold interventions that might be well-suited for stigmatized conditions such as loneliness. Recently, guided ICBTs has produced promising results in alleviating loneliness (Käll et al., 2021; Käll et al., 2020). In our research group, we evaluated the efficacy of ICBT for loneliness with guidance (i.e., weekly written individualized feedback from a coach) or automated messages (i.e., weekly non-individualized feedback) (Seewer et al., 2023). Overall, participants in both intervention groups showed lower loneliness scores compared to the waitlist condition, and participants in the guided condition scored lower on loneliness than in the automated message condition at post-treatment. However, not all participants benefited equally from the intervention. In total, 52.0 % of participants in the guided and 58.8 % in the automated messages condition showed no reliable improvement in loneliness or deteriorated from pre to post-assessment (Seewer et al., 2023). Therefore, identifying who profits from ICBT with or without guidance is crucial as this would provide information for whom such an intervention might be indicated.

### 1.3 Predictors of face-to-face loneliness intervention

Non-specific predictors and moderators can be examined to identify individuals who might benefit from a particular intervention. The first describes baseline variables that predict the outcome irrespective of treatment condition, i.e., main effects of predictor variables (Kraemer et al., 2002). With moderator variables, the effect of the intervention groups on the outcome differs depending on the level of the moderator which necessitates the inspection of the interaction *group* × *moderator variable* (Kraemer et al., 2002). Findings regarding predictors or moderators of loneliness interventions are limited to one study reporting on two trials in which a face-to-face group-based intervention was administered and predictors of treatment response were examined (Cruwys et al., 2022). Overall, higher loneliness, social anxiety, or depression scores at baseline, fulfilling a psychiatric diagnosis, or attending more sessions predicted greater improvement in loneliness (Cruwys et al., 2022). This study provided the first evidence on who might benefit from a group-based face-to-face loneliness intervention. However, it may be that other processes lead to therapeutic change in face-to-face vs. Internet-based cognitive behavioral treatment (Donker et al., 2013). Consequently, variables predicting treatment effects of loneliness interventions in an individual online setting, i.e., ICBT, need to be evaluated.

### 1.4 Predictors and moderators of ICBTs with and without guidance

A recent systematic review synthesized findings from prediction studies on guided ICBTs across various psychological disorders (Haller et al., 2023). Overall, no demographic variables consistently predicted outcome. On the other hand, treatment-related variables, such as adherence or working alliance, predicted better outcomes, while clinical variables, such as higher baseline symptom severity, predicted worse treatment outcome but better treatment response. In addition, a tendency for less favorable treatment outcomes in the presence of comorbid depressive and anxiety symptoms was observed (Haller et al., 2023). However, this review only focused on guided ICBT and did not include studies assessing predictors / moderators of ICBT with automated messages. Thus, from this review it cannot be concluded whether the same variables predict the outcome of ICBT with human guidance or automated messages.

There is limited evidence that the same baseline variables are associated with treatment outcome in both forms of ICBT equally. For example, in a study examining the efficacy of self-guided and therapist-guided Internet-based treatment of social phobia, measures of program usage, i.e., time spent within the program or number of modules completed, were associated with treatment outcome in both treatment conditions (Berger et al., 2011). Conversely, adherence and symptom severity at baseline were predictors of diagnosis-free status post-treatment of an Internet-based intervention for social anxiety disorder in the guided, but not in the self-guided condition (Nordgreen et al., 2012). Moreover, differential effects of baseline severity on the efficacy of ICBT with or without guidance were identified for depression, suggesting a more favorable treatment outcome in the guided compared to the self-guided condition for participants with increased baseline symptom severity (Karyotaki et al., 2021). While predictors / moderators of treatment outcome might differ depending on the support provided with the ICBT, to determine who benefits from an ICBT either with guidance or automated messages might be particularly relevant for alleviating loneliness. In addition to an increase in adherence, guidance might provide lonely individuals with experiences similar to those found in social relationships, like validation or empathy, aspects that lonely individuals may lack in their social relationships (Käll et al., 2020).

In addition to predictors / moderators of treatment outcome previously examined, it is important to investigate if loneliness-specific aspects such as increased feelings of loneliness, duration of loneliness, satisfaction with quality of social relationships or clinical variables like childhood trauma, that affects the experience of safe and caring relationships (de Heer et al., 2022) are associated with the outcome of an ICBT with guidance or automated messages. Overall, the question remains for whom ICBT with guidance or automated messages is beneficial and if predictors and moderators of outcome in previous studies on various psychological disorders (Haller et al., 2023) are also valid for ICBT for loneliness. This would allow ICBT for loneliness with guidance or automated messages to be offered more specifically to those who would benefit from it, and interventions to be adapted for those who do not.

### 1.5 Purpose of the present study

In this study, we investigated if demographic, clinical, loneliness-specific, or treatment-related (baseline-) variables moderate the treatment effects in ICBT for loneliness with guidance (GU) or automated messages (AM) compared to a waitlist-control group at post-assessment. Furthermore, non-specific predictors and moderators of outcome at post-treatment were examined across intervention conditions to identify individuals who might respond better to an ICBT for loneliness (regardless of support format). The analyses were considered exploratory since evidence regarding moderating and non-specific predictive variables of loneliness interventions is limited and lacking completely concerning ICBT for loneliness. Thus, no specific hypotheses were formulated. Potential predictor and moderator variables were selected based on theoretical assumptions and empirical findings from the literature on loneliness as well as Internet-based interventions.

## 2 Method

### 2.1 Trial design

This secondary analysis used data from a three-arm randomized controlled trial (RCT) (Seewer et al., 2023). Eligible participants were randomly assigned to ICBT with guidance, to ICBT with automated messages, or to a waitlist-control group. While intervention groups received immediate access to the intervention after randomization, the waitlist-control group got access 10 weeks after randomization. The RCT was preregistered on clinicaltrials.gov (NCT04655196, registration date: 07/12/2020), conducted in accordance with the declaration of Helsinki, and received approval by the Cantonal Ethics Committee Bern (CEC; ID: 202–01298). A study protocol was published (Seewer et al., 2022), and the efficacy of the ICBT will be reported elsewhere (Seewer et al., 2023). All subjects provided signed informed consent before participating in the trial.

### 2.2 Procedure and participants

In total, 243 participants were recruited from the general population in German-speaking countries from May 2021 to July 2022. Participants were recruited via social media, articles/interviews in newspapers/radio, Google ads, newsletters, the study website, and the website from our research hub. The inclusion criteria were the following: ≥ 18 points on the UCLA Loneliness Scale – 9 item version (UCLA-9; Luhmann et al., 2016), ≥ 18 years old, sufficient German language skills, possibility to access the Internet, and a contact in case of emergency. The exclusion criteria were the following: current moderately severe or severe depressive symptoms (PHQ-9 ≥ 14), lifetime diagnosis of psychotic or bipolar disorder, current severe substance use disorder, or acute suicidal plans. After the baseline assessment, randomized participants completed questionnaires at post-assessment (i.e.,10 weeks). For more details see (Seewer et al., 2023).

In total, 180 of the 243 randomized participants completed the UCLA-9 at baseline and post-assessment, and their data were used to investigate moderators and non-specific predictors of treatment outcome at post-assessment. Overall, the sample was mainly female (*n* = 143, 79.4 %), single (*n* = 141, 78.3 %), had a university degree (*n* = 115, 64.3 %) and was 47.2 (*SD* = 14.5) years old. Baseline characteristics overall and by group for the completer sample are displayed in Table S1 in the Online Supplementary Material.

### 2.3 Description of intervention

The Internet-based self-help program SOLUS-D is an adapted version of an ICBT for loneliness developed and evaluated in Sweden (Käll et al., 2020). SOLUS-D consists of 9 modules based on CBT principles, self-compassion, and mindfulness. The content was mainly text- but also video- and audio-based. Practical exercises and a diary function were implemented to practice and integrate theoretical input into everyday life and to facilitate a change of perspective. On average, it takes about 50 min to complete one module, and we recommended working on one module per week. Participants were free to spend more time implementing the exercises or to repeat the content. A more detailed description of the intervention is provided in the study protocol (Seewer et al., 2022).

### 2.4 Study conditions

Participants were randomly allocated to one of two intervention groups or a waitlist-control group with a ratio of 2:2:1. Participants in the *SOLUS-D + Guidance (GU)* had access to the ICBT and received weekly semi-standardized feedback from trained and supervised coaches within the self-help platform. The personalized part of the messages entailed feedback on participants' work during the previous week and answers to their questions. Individuals in the *SOLUS-D + Automated Messages (AM)* condition had access to the self-help program but received weekly, fully standardized messages to motivate continuous engagement with the intervention. The *waitlist-control group (WL)* received access to the intervention upon completing the post-assessment ten weeks after randomization.

### 2.5 Outcome measure

#### 2.5.1 Loneliness at endpoint

The UCLA-Loneliness scale – 9 item version (UCLA-9; Luhmann et al., 2016) was used to assess loneliness as an outcome at post-assessment. The nine items are rated on a 4-point Likert scale ranging from never (1) to always (4). A sum score ranging from 9 to 36 was calculated, with higher scores indicating higher levels of loneliness.

#### 2.5.2 Reliable improvement of loneliness

The second outcome measure was change in loneliness operationalized as reliable improvement on the UCLA-9 (Luhmann et al., 2016) from baseline to post-assessment. The Reliable Change Index was calculated according to the formula by Jacobson and Truax (1991), and we used Cronbach's alpha (0.90) from a sample of the general population of German-speaking countries (*n* = 813, unpublished data). Standard deviations were calculated for each subsample separately. Consequently, a reduction of 2.73 (WL vs. GU), 2.92 (WL vs. AM), or 2.76 (AM vs. GU) from pre to post was considered as reliable improvement (reliable improvement = 1, no reliable improvement = 0).

### 2.6 Potential predictor and moderator variables

We grouped 28 potential predictor and moderator variables into four thematic domains: socio-demographic variables, clinical variables, loneliness-related variables, and treatment-related variables (intervention groups only). All variables were collected before randomization, except for treatment-related variables, which were collected five (working alliance) and ten weeks (numbers of modules accessed and time spent within the program) after randomization, respectively. Participants completed all questionnaires online via the survey platform Qualtrics. Current psychiatric diagnoses were assessed with a diagnostic interview (Margraf et al., 2017) via phone.

#### 2.6.1 Socio-demographic variables

We assessed age, gender (male = 0, female = 1, other = 2), education (no university degree = 0, university degree = 1), living situation (alone = 0, with others = 1), relationship status (no relationship = 0, in a relationship = 1), and employment situation (no paid work/ unemployed = 0, paid work = 1) as potential socio-demographic predictor/moderator variables.

#### 2.6.2 Psychological distress variables

At baseline, participants indicated their current use of psychological treatment (no treatment = 0, in treatment = 1) and psychotropic medication (no medication = 0, medication = 1). The absence (0) or presence (1) of one or more psychiatric diagnoses according to the diagnostic interview (Mini-DIPS OA; Margraf et al., 2017) was binary coded. Additionally, we administered self-report measures to assess depressive symptoms (PHQ-9; Kroenke et al., 2001; Löwe et al., 2004), social anxiety (SIAS-6& SPS-6; Peters et al., 2012), and maladaptive personality traits (PID5BF+; Kerber et al., 2022). The five subscales of the Childhood Trauma Questionnaire (CTQ; Bernstein et al., 2003; Wingenfeld et al., 2010) were used to indicate if participants had experienced none-moderate (0) or moderate-extreme (1) emotional abuse, physical abuse, sexual abuse, emotional neglect, and physical neglect. The CTQ scores of each subscale were dichotomized according to the assessment of severity of maltreatment in Häuser et al. (2011).

#### 2.6.3 Loneliness related variables

Duration of loneliness assessed at baseline was dichotomized to reflect chronic loneliness (i.e., > 2 years, coded as 1) and shorter periods of loneliness (coded as 0). No agreed-upon critical duration of chronic loneliness exists. Therefore, we followed Young's (1982) definition of loneliness as chronic if it lasts at least two years. Two single items were administered to assess the satisfaction with the quality or quantity of social relationships, i.e., “*How satisfied are you with the number of social relationships you currently have?*” and “*How satisfied are you with the quality of social relationships you currently have?*” They were rated on a 4-point Likert scale ranging from 1 (very unsatisfied) to 4 (very satisfied). Suffering caused by loneliness was assessed with the single item “*How much do you feel burdened by the feelings of loneliness indicated?)* and was rated on a 5-point Likert scale ranging from 0 (“not at all”) to 4 (“very much”). The three subscales of the Social Network Index (SNI; Cohen et al., 1997) were used to measure social isolation with the subscale *social network size*, the diversity of social networks, and the embeddedness within the social networks.

#### 2.6.4 Treatment-related variables

The number of modules accessed (ranging from 0 to 9) and time spent within the program (in minutes) were recorded via the platform on which the self-help program was provided. Furthermore, the subscale “task & goals” from the working alliance inventory was assessed at mid-treatment (5 weeks after randomization) with the Working-Alliance Inventory for guided Internet interventions (WAI—I; Gómez Penedo et al., 2020).

### 2.7 Data analysis

Statistical analyses were performed using R Statistical Software (v4.2.1, R Core Team, 2022) and R Studio (v2023.6.1.524, Posit team, 2023). Specifically, the following R packages were mainly used for the analyses: *car* (Fox and Weisberg, 2019), *lm.beta* (Behrendt, 2023), *emmeans* (Lenth, 2022), and *performance* (Lüdecke et al., 2021). Descriptive analyses were conducted to compare baseline differences between study conditions and participants with and without missing outcome data. Continuous variables were analyzed with ANOVAs or *t*-tests, and categorical variables with χ^2^ -tests. To identify predictors and moderators of treatment outcome and response at post-assessment, we followed the approach advocated by Kraemer et al. (2002), which was administered in previous studies (e.g., Fournier et al., 2009). In this framework, the interaction between predictor variables of interest and treatment condition is investigated. With a significant interaction, the predictor is termed *moderator*, signifying varying treatment effects based on the predictor's value. If the interaction is non-significant, but a lower-order term is, the variable is termed a non-specific predictor indicating a significant effect on the outcome irrespective of treatment condition (Fournier et al., 2009).

Multiple linear regression models were estimated for loneliness as an endpoint (UCLA-9 at post-assessment), and multiple logistic regression models were estimated for the categorical outcome (reliable improvement at post-assessment). The analyses were conducted separately in the WL vs. GU, WL vs. AM, and AM vs. GU samples. Since we examined various predictor variables, which necessitates many statistical tests, we applied the method proposed by Fournier et al. (2009), which is a way to balance out Type-I and Type-II errors. First, we estimated models for each group of predictor variables described above and applied a stepwise procedure within each group. In step 1, we examined if the model containing all predictors was significant. In step 2, we entered all predictors into the model with significant values of *p* < 0.20 from step 1. In step 3, all significant variables from step 2 with *p* < 0.10 were retained. Finally, all significant predictors at *p* < 0.05 in step 3 were kept in the model in step 4. Once all predictor variables from all predictor groups were identified (significant at *p* < 0.05 in step 4), they were simultaneously entered into a final model. This allowed us to examine the effects of all predictors while controlling for the other predictor variables. All continuous variables were grand-mean centered, and dichotomous variables were set to 0 and 1.

The analyses were performed in the complete-case sample and adjusted for baseline variables significantly associated with missing at either post-assessment. Missing predictor variables were not imputed. Adjusting for baseline covariates associated with missingness was chosen because this procedure can yield comparable estimates to other strategies for handling missing outcome data, i.e., multiple-imputation (Groenwold et al., 2012). Completers and non-completers of the post-assessment significantly differed regarding age and condition (*p*'s < 0.01) (see Table S2 in the Online Supplementary Materials). Despite significant differences between completer and non-completer at post-assessment regarding *number of modules accessed* and time spent within the program (*p*'s < 0.001), we did not adjust for those variables because they were assessed post-randomization.

The alpha level was set to 0.05 for all analyses. Corrections for multiple comparisons were not applied because of the exploratory nature of this study (Bender and Lange, 2001). Standardized beta is reported to facilitate the comparability of the coefficients in the linear regression models. Odds ratios [Exp(B)] are reported as an effect size for the logistic regression models. Confidence intervals for the effect size estimates are reported where applicable.

## 3 Results

### 3.1 Descriptive statistics and dropout analysis

Pre-treatment levels of predictor variables did not significantly differ between conditions in the completer sample at post-assessment (all *p*'s > 0.97) (see Table S1 in the Online Supplementary Materials). The completer sample consisting of both intervention groups significantly differed concerning time spent within the program (*p* = 0.02). Only variables with significant baseline differences between conditions were entered into the models as covariates, but not variables assessed post-randomization.

In total, 180 participants completed the post-assessment, corresponding to a pre-post dropout rate of 25.9 %. Dropout significantly differed between study conditions, χ^2^(2) = 15.50; *p* < 0.001, with GU = 28.6 %, AM: 34.0 %, and WL = 4.2 % non-completers.

### 3.2 Predictors and moderators: level of loneliness as outcome

Multiple linear regression models were estimated to assess non-specific predictor (intervention groups only) and moderator variables of the UCLA-9 score at post-assessment. All models were adjusted for age and condition because of differences between completer and non-completer. Additionally, we accounted for the UCLA-9 at baseline except in the models with loneliness-specific variables because baseline loneliness was already being examined as a potential predictor or moderator. The final model was adjusted for the covariates and the UCLA-9 baseline score if the latter did not emerge as a significant predictor/moderator.

##### 3.2.1 Waitlist-control group vs. SOLUS-D + guidance

In step 4, no interactions were statistically significant at *p* < 0.05 in any predictor domain. Thus, a final model was not estimated.

##### 3.2.2 Waitlist-control group vs. SOLUS-D + automated messages

In step 4, no interactions were statistically significant at p < 0.05 in any predictor domain. Thus, no final model was estimated.

##### 3.2.3 Automated messages vs. guidance

In step 4 of the domain-specific models, the UCLA-9 baseline score and working alliance were significant at *p* < 0.05. The final model, including the covariates, explained 39.1 % of the variance in the UCLA-9 score at post-assessment. The UCLA-9 baseline score, working alliance, and the covariate age were identified as significant non-specific predictors of loneliness at post-assessment. Participants experiencing a better working alliance scored lower on loneliness at post-assessment across intervention conditions. In contrast, higher loneliness and age at baseline were associated with higher loneliness scores at post-assessment when accounting for the other variables. Table 1 displays the results of the final linear regression model.

**Table 1:** Multiple Linear Regression Analysis: Final Model for Treatment Outcome at Post-assessment (UCLA-9 Loneliness Scale).

	Post-assessment					
	AM vs. GU					
	*B*	*SE*_*B*_	*β*	*LCI*	*UCI*	*p*
Intercept	21.26	0.40	N/A	20.47	22.05	<0.001
UCLA-9 pre^a^	0.69	0.09	0.56	0.52	0.86	<0.001
Condition^b^	−0.55	0.55	−0.07	−1.64	0.53	0.32
Age^a^	0.04	0.02	0.14	0.00	0.08	0.04
WAI_TG^a^	−0.95	0.36	−0.19	−1.65	−0.24	<0.01
N of Obs.	129					
*R*^2^	0.41					
Adjusted *R*^2^	0.39					
Statistic	*F*(4,124) = 21.53 *p* < 0.001					

*Note.* AM = SOLUS-D + automated messages, GU = SOLUS-D + guidance. UCLA-9 = University of California Loneliness Scale–9 item version; WAI_TG = Working Alliance Inventory – Subscale task & goal.

a Continuous variables were centered at the grand mean.

b Reference group is AM. Condition, therefore, describes the estimate for the GU condition.

### 3.3 Predictors and moderators: reliable improvement in loneliness as outcome

We estimated multiple logistic regression models separately in the WL vs. GU and WL vs. AM samples to assess moderator variables of treatment response at post-assessment. Further multiple logistic regression models were estimated to identify non-specific predictors and moderators of treatment response across the active intervention conditions at post-assessment. The models with the socio-demographic, clinical, and treatment-related variables were adjusted for the UCLA-9 baseline score, age, and condition. Since the UCLA-9 was assessed as a potential predictor or moderator in the models with the loneliness-specific variables, adjustments were only made for age and condition. The final model was adjusted for the covariates and the UCLA-9 baseline score if the latter did not emerge as a significant predictor/moderator.

##### 3.3.1 Waitlist-control group vs. SOLUS-D + guidance

At step 4 of the models within predictor domains, the interactions education × condition and suffering from loneliness × condition were significant at *p* < 0.05. They were included in the final model with the covariates. The final model was statistically significant, χ^2^(7) = 20.78, *p* < 0.001, with a Nagelkerke's *R*^2^ of 0.22. Table 2 displays the results of the final model. The interaction between education level and condition was not statistically significant, but suffering from loneliness emerged as a significant moderator in the final model. Subsequent contrast analyses revealed significantly lower odds of reliable improvement for participants in the WL than the GU condition at −1 *SD* (*OR* = 0.12, 95 %-CI [0.03;0.48], *p* < 0.01) and at the mean (*OR* = 0.30, 95 %-CI [0.13; 0.74], *p* < 0.01) of suffering from loneliness.

**Table 2:** Multiple Logistic Regression Analysis: Final Models for Treatment Response at Post-assessment (i.e., Reliable Improvement on the UCLA-9 Loneliness Scale).

	Post-assessment					
	WL vs. GU			AM vs. GU		
	*OR*	*95%CI*	*p*_*OR*_	*OR*	*95%CI*	*p*_*OR*_
Intercept	0.78	0.28–2.14	0.62	0.99	0.60–1.65	0.98
UCLA-9 pre^a^	0.99	0.86–1.15	0.93	1.08	0.97–1.21	0.18
Condition^b^	1.40	0.37-5.47	0.62	1.55	0.77–3.15	0.23
Age^a^	0.97	0.94-1.00	0.06	0.98	0.95–1.00	0.08
Lsuffer ^a^	1.89	0.80–4.79	0.16			
Education	0.30	0.07–1.16	0.08			
Lsuffer * condition^b^	0.33	0.10–0.96	0.046			
Education * condition^b^	5.56	0.99–31.51	0.05			
N of Obs.	115			134		
Nagelkerkers *R*^2^	0.22			0.06		
Hosmer-Lemeshow-Test for goodness of fit	*χ*^2^(8) = 4.46, *p* = 0.81			*χ*^2^(8) = 7.73, *p* = 0.46		

*Note.* WL = Waitlist-control group, AM = SOLUS-D + automated messages, GU = SOLUS-D + guidance. UCLA-9 = University of California Loneliness Scale–9 item version; Lsuffer = Subjective burden of feelings of loneliness.

a Continuous variables were centered at the grand mean.

b Reference group is WL when compared to intervention groups and AM when intervention groups are compared against each other. Condition, therefore, describes the estimate for the non-reference category.

##### 3.3.2 Waitlist-control group vs. SOLUS-D + automated messages

At step 4 of the models within predictor domains, no statistically significant interactions at *p* < 0.05 emerged. Thus, no final model was estimated.

##### 3.3.3 Automated messages vs. guidance

No significant predictor variables remained at step 4 of the multiple logistic regression models within predictor domains. Moreover, the final model containing the covariates was not significant, χ^2^(3) = 6.64, *p* = 0.08, with a Nagelkerke's *R*^2^ of 0.06. Table 2 displays the results of the final model.

## 4 Discussion

This exploratory study examined a large number of baseline variables as predictors and moderators of treatment outcome and response after the intervention phase of an ICBT for loneliness using data from an RCT (Seewer et al., 2023). While suffering from loneliness moderated treatment response for ICBT with guidance versus the WL, no variables were identified that moderated the outcome or treatment response in the AM versus the WL condition. Also, increased levels of loneliness and higher age positively predicted treatment outcome (i.e., higher loneliness scores) for ICBT with guidance and automated messages. Furthermore, a better fit between participants' needs and the tasks and goals of the intervention was associated with better treatment outcome across intervention conditions. However, no significant moderator of the treatment effect of ICBT with guidance or automated messages emerged. Since the present analyses were exploratory in nature, and the relatively small sample size could have affected the results, the findings of the study must be interpreted with caution.

Individuals in the GU who suffered less from their feelings of loneliness showed a better treatment response than the WL, but no differences between intervention and waitlist-control condition emerged in those heavily burdened by their feelings of loneliness. Often, individuals who feel lonely experience a low sense of control and self-efficacy (Heinrich and Gullone, 2006). Thus, individuals who suffer more from their feelings of loneliness might doubt their ability to change their condition through their efforts. Consequently, the transfer of the intervention content to everyday life might be limited, lowering the likelihood of improvement in loneliness. Therefore, inducing hope for change and offering easy exercises at the beginning of the intervention, which quickly lead to initial successes, might be essential to increase motivation and self-efficacy. Yet, further studies are needed to follow-up on this assumption.

Increased baseline loneliness predicted higher loneliness scores at post-assessment but was unrelated to treatment response. This finding partially aligns with prior evidence, e.g., in guided ICBTs, suggesting worse treatment outcome (i.e., higher post- or FU score) but better treatment response (i.e., greater symptom reduction) in participants with higher baseline symptom severity (Haller et al., 2023). Also, contrary to our findings, in a face-to-face group-based loneliness intervention, higher baseline loneliness was associated with greater improvements in loneliness (Cruwys et al., 2022). It should be mentioned that the baseline levels of loneliness of the participants in Cruwys et al. (2022) were comparably lower than in our sample (Seewer et al., 2023). Additionally, outcome measures were assessed at a 4-month follow-up (i.e., two months after the end of the intervention) (Cruwys et al., 2022) and not at the end of the intervention as in our study. However, group interventions might offer the advantage of facilitating new social connections and thus lead to more pronounced reductions in loneliness. Nonetheless, the group setting could challenge lonely individuals, particularly those who hold strong negative cognitions about themselves and others (Cacioppo and Hawkley, 2009). Thus, they might not seek such an intervention. Future research should investigate if building new contacts is essential for better treatment response, especially in severely lonely individuals. To maintain low-threshold access to the intervention, a first step might be incorporating a discussion forum in the self-help platform. This would still include those for whom a face-to-face group is a barrier but offer a platform for exchange with others.

Also, older age was associated with missing outcome assessment and higher loneliness scores at post-assessment across intervention conditions. Similar findings have been observed in other studies, e.g., for adults with generalized anxiety disorder (Edmonds et al., 2018). The higher missing rate for younger adults might have influenced our results. Future studies are warranted to assess why younger adults drop out of ICBTs more often and how to counteract this phenomenon. Furthermore, it is also possible that several risk factors, such as poorer physical health or widowhood (Barjaková et al., 2023), which might be more common among older people, contribute to higher loneliness scores after an ICBT for loneliness. In this respect, further studies might investigate whether an age-specific adaptation of the intervention, i.e., addressing age-specific risk factors more specifically, increases the effects of ICBT in alleviating loneliness, particularly in older individuals.

Finally, individuals who perceived a good fit between the tasks and goals of the intervention and their own needs benefited from the ICBT, irrespective of the intervention condition. This aligns with previous work on the possibility and importance of establishing a good working alliance in Internet-based interventions (Flückiger et al., 2018; Kaiser et al., 2021). Thus, ICBTs for loneliness might benefit those who feel confident enough to work on their feelings of loneliness autonomously and at their own pace.

### 4.1 Limitations

This study has limitations to consider. First, the study was not powered for predictor and moderator analyses. Thus, the limited sample size might have hindered the detection of small effects and led to imprecise coefficient estimations, as signified by large confidence intervals. The results should thus be interpreted cautiously. Despite the exploratory nature of our analyses, they provide valuable insights into potential predictor or moderator variables. These findings could inform future studies with larger sample sizes to replicate and further investigate these relationships (Kraemer et al., 2002). Second, to control for statistical artifacts, e.g., regression to the mean, our analyses were conducted for the short-term outcome. This, however, hinders us from drawing conclusions regarding predictors and moderators of long-term outcome, which should be examined in future studies. Third, the sample of the randomized controlled trial was self-selected and thus consisted of individuals who presumably were open to and motivated to use an Internet-based intervention. The generalizability of the results to other samples is therefore limited, and future studies should investigate whether the predictors and moderators found are valid in other settings, such as primary care. Fourth, participants with moderately severe and severe depressive symptoms were excluded from to study for ethical reasons. Therefore, the findings cannot be applied to participants with more strongly pronounced depressive symptoms and further investigations in clinical samples are warranted. Fifth, the measures of some variables investigated as predictor variables (i.e., depressive symptom, loneliness) were used to assess eligibility for study inclusion or exclusion respectively. Thus, the range of those baseline variables was restricted, and it cannot be ruled out that this restriction in range has affected the results of the analyses.

### 4.2 Conclusion

The results of the current trial provide preliminary evidence that some baseline characteristics, such as the subjective burden of loneliness, level of loneliness, age, and a better match regarding the task and goals of the intervention with the participants' needs, predict treatment outcome and response to ICBT for loneliness. Guided ICBT might be particularly helpful for those with a lower perceived burden of their feelings of loneliness. Also, people who feel less lonely, are younger, or feel confident enough to autonomously engage via an Internet-based intervention with their thoughts, feelings, and behaviors regarding their loneliness might benefit from either ICBT with guidance or automated messages. Conversely, the absence of further moderators, e.g., duration of loneliness or childhood trauma, may indicate that ICBT with guidance or automated messages could be similarly effective for individuals with different baseline characteristics. Therefore, personal preferences of those affected by loneliness might be considered when choosing between ICBT with guidance or automated messages. Furthermore, this finding also points to the limited understanding of the underlying mechanisms of human guidance in relation to the effectiveness of Internet-based self-help interventions. It thus highlights the need for more research in this area. Overall, adjustments to the ICBT for individuals who feel subjectively more burdened due to loneliness, have higher baseline loneliness scores or are older could lead to greater reductions in the overall burden of loneliness. Since this was not a confirmatory study, the results still need to be replicated in further studies with larger samples.

## CRediT authorship contribution statement

T.K., T.B., and G.A. designed the trial, and T.K. received funding for this trial. A.K. and G.A. provided the Swedish version of the Internet-based cognitive-behavioral intervention program. N.S., A.S., and T.K. designed and extended the German version of the Internet-based intervention. N.S., A.S., and T.K. conducted the RCT. N.S. and T.K. conceptualized the methods of the secondary analysis. N.S. analyzed the results. N.S. and T.K. wrote the first draft of the manuscript. All authors contributed to the final version of the manuscript.

## Declaration of competing interest

The authors declare no competing interests regarding the publication of this paper.

## Data Availability

De-identified data and statistical codes supporting the findings of this study are available upon publication of the manuscript on OSF (https://osf.io/7m5nr/).

## Appendix A Supplementary data

Supplementary material Image 1
