# Supplementary material for: Predictors and moderators of outcome of ICBT for loneliness with guidance or automated messages - A secondary analysis of a randomized controlled trial
Source: Internet Interv. 2023 Dec 18;35:100701. doi: 10.1016/j.invent.2023.100701 (PMC10772709; doi:10.1016/j.invent.2023.100701)
Supplement: Supplementary file 1 — Supplementary material [file mmc1.docx]

Supplementary Materials

Predictors and moderators of outcome of ICBT for loneliness with guidance or automated messages - A secondary analysis of a randomized controlled trial

Table of Content

Methods 3

Procedure and Participants 3

Table S1 3

Statistical Analyses 5

Table S2 5

References 6

# Methods

## Procedure and Participants

### Table S1

*Predictors at Baseline and Outcome Measures, Overall and Divided by Group for the Completer Sample at Post-assessment*

|  | Total  (n = 180) | GU  (n = 70) | AM  (n = 64) | WL  (n = 46) | Statistic |
| --- | --- | --- | --- | --- | --- |
| **Socio-demographic variables** |  |  |  |  |  |
| Age, *M* (*SD*) | 47.23 (14.45) | 47.06 (14.81) | 49.00 (14.16) | 45.04 (14.29) | *F* (2,177) = 1.01; *p* = .37 |
| Female, *n* (%) | 143 (79.44) | 55 (78.57) | 51 (79.69) | 37 (80.43) | *X*^2^(4) = 1.39; *p* = .85 |
| In a relationship, *n* (%) | 39 (21.67) | 17 (24.29) | 10 (15.63) | 12 (26.09) | *X*^2^(2) = 2.19; *p* = .33 |
| Living alone, *n* (%) | 119 (66.11) | 48 (68.57) | 47 (73.44) | 24 (52.17) | *X*^2^(2) = 5.71; *p* = .06 |
| University, *n* (%) | 115 (64.25) | 47 (68.12)^a^ | 39 (60.94) | 29 (63.04) | *X*^2^(2) = 0.78; *p* = .68 |
| Paid work, n (%) | 116 (65.17) | 43 (61.43) | 41 (65.08)^a^ | 32 (71.11)^a^ | *X*^2^(2) = 1.13; *p* = .57 |
| **Clinical Variables** |  |  |  |  |  |
| Current psychological treatment (yes), *n* (%) | 61 (33.89) | 23 (32.86) | 23 (35.94) | 15 (32.61) | *X*^2^(2) = 0.19; *p* = .91 |
| Current medication (yes), *n* (%) | 32 (17.78) | 10 (14.29) | 12 (18.75) | 10 (21.74) | *X*^2^(2) = 1.12; *p* = .57 |
| Current psychiatric diagnosis, *n* (%) | 98 (54.44) | 39 (55.10) | 36 (57.73) | 23 (50.00) | *X*^2^(2) = 0.50; *p* = .78 |
| Depressive symptoms (PHQ-9), *M* (*SD*) | 8.74 (3.26) | 9.13 (3.21) | 8.42 (3.33) | 8.61 (3.24) | *F* (2,177) = 0.83; *p* = .44 |
| Social Anxiety (SPS-6), *M* (*SD*) | 3.22 (3.62) | 3.24 (3.19) | 3.12 (4.03) | 3.30 (3.71) | *F* (2,177) = 0.04; *p* = .97 |
| Social Interaction Anxiety (SIAS-6), *M* (*SD*) | 5.37 (4.13) | 5.37 (4.12) | 5.73 (4.31) | 5.98 (3.94) | *F* (2,177) = 0.32; *p* = .73 |
| Personality Inventory (PID5BF+), *M* (*SD*) | 1.03 (0.29) | 0.99 (0.29) | 1.06 (0.29) | 1.04 (0.30) | *F* (2,177) = 1.10; *p* = .33 |
| Childhood Trauma (CTQ)^c^ |  |  |  |  |  |
| Emotional abuse, *n* (%) | 85 (47.22) | 33 (47.14) | 32 (50.00) | 20 (43.47) | *X*^2^(2) = 0.46; *p* = .80 |
| Physical abuse, *n* (%) | 26 (14.44) | 10 (14.29) | 12 (18.75) | 4 (8.70) | *X*^2^(2) = 2.19; *p* = .33 |
| Sexual abuse n, (%) | 38 (21.11) | 14 (20.00) | 15 (23.44) | 9 (19.57) | *X*^2^(2) = 0.33; p = .85 |
| Emotional neglect, *n* (%) | 97 (53.89) | 32 (45.71) | 39 (60.94) | 26 (56.52) | *X*^2^(2) = 3.29; *p* = .19 |
| Physical neglect, *n* (%) | 65 (36.11) | 23 (32.86) | 23 (35.94) | 19 (41.30) | *X*^2^(2) = 0.86; *p* = .65 |
| **Loneliness specific variables** |  |  |  |  |  |
| Loneliness at baseline (UCLA-9), *M* (*SD*) | 24.13 (3.20) | 23.83 (2.98) | 24.53 (3.33) | 24.04 (3.35) | *F* (2,177) = 0.83; *p* = .44 |
| Duration of loneliness > 2 years, *n* (%) | 158 (89.77) | 59 (85.51)^a^ | 56 (90.32)^b^ | 43 (95.56)^a^ | *X*^2^(2) = 3.03; *p* = .22 |
| Suffering from loneliness | 2.26 (0.84) | 2.16 (0.79) | 2.38 (0.91)^a^ | 2.26 (0.83) | *F* (2,176) = 1.17; *p* = .31 |
| Suffering from loneliness (dichotomized) , *n* (%) | 148 (75.90) | 55 (78.57) | 55 (87.30)^a^ | 38 (82.61) | *X*^2^(2) = 1.77; *p* = .41 |
| Satisfaction with quantity of social relationships | 1.88 (0.77) | 1.97 (0.72) | 1.73 (0.76) | 1.93 (0.83) | *F* (2,177) = 1.78; *p* = .17 |
| Satisfaction with quality of social relationships | 2.28 (0.82) | 2.31 (0.84) | 2.22 (0.83) | 2.30 (0.79) | *F* (2,177) = 0.26; *p* = .77 |
| Social isolation (SNI), *M* (*SD*) |  |  |  |  |  |
| Network size | 11.05 (6.06) | 10.69 (5.00) | 11.03 (6.80) | 11.63 (6.51) | *F* (2,177) = 0.34; *p* = .72 |
| Network embeddedness | 1.58 (0.95) | 1.44 (0.85) | 1.58 (1.07) | 1.78 (0.92) | *F* (2,177) = 1.79; *p* = .17 |
| Network diversity | 3.93 (1.50) | 3.99 (1.44) | 3.95 (1.66) | 3.80 (1.39) | *F* (2,177) = 0.21; *p* = .81 |
| **Treatment related variables**^e^ |  |  |  |  |  |
| Modules accessed, *M* (*SD*) | 6.42 (2.92) | 7.73 (1.70) | 7.47 (2.32) | - | *t(132) = -0.74; p = .46* |
| Time spent within the program (min), *M* (*SD*) | 467.40 (462.48) | 677.83 (588.97) | 484.86 (338.82) | - | *t(132) = -2.97; p = .02* |
| Working Alliance – Task&Goal (WAI), *M* (*SD*) | 3.20 (0.75) | 3.27 (0.73)^a^ | 3.10 (0.82)^c^ | - | *t(127) = -1.24; p = .22* |
| **Outcome variables** |  |  |  |  |  |
| UCLA-9, *M* (*SD*) | 21.59 (4.05) | 20.04 (3.73) | 21.84 (4.01) | 23.04 (4.10) | *F* (2,177) = 6.50; *p* < .01 |
| Reliable Improvement (yes), *n* (%) | 88 (48.89) | 42 (60.00) | 32 (50.00) | 14 (30.43) | *X*^2^(2) = 9.76; *p* < .01 |

*Note.* GU = SOLUS-D with guidance; AM = SOLUS-D with automated message; WL = waitlist control group.

^a^ *n* of missings = 1

^b^ *n* of missings = 2

^c^ *n* of missings = 4

^d^ Participants with scores rated as “moderate to severe” or “severe to extreme” according to the assessment of severity of maltreatment (Häuser et al., 2011)

^e^ Intervention conditions only.

## Statistical Analyses

### Table S2

*Predictors at Baseline for the Completer and Non-completer sample at Post-assessment*

|  | Completer  (n = 180) | Non-Completer  (n = 63) | Statistic |
| --- | --- | --- | --- |
| **Socio-demographic variables** |  |  |  |
| Condition, n (%) |  |  | *X*^2^(2) = 15.50; *p* < .001 |
| GU | 70 (38.89) | 28 (44.44) |  |
| AM | 64 (35.56) | 33 (52.38) |  |
| WL | 46 (25.56) | 2 (3.17) |  |
| Age, *M* (*SD*) | 47.23 (14.45) | 41.60 (15.31) | *t(241) = 2.62; p < .01* |
| Female, *n* (%) | 143 (79.44) | 48 (76.19) | *X*^2^(2) = 1.20; *p* = .55 |
| In a relationship, *n* (%) | 39 (21.67) | 16 (25.40) | *X*^2^(1) = 0.19; *p* = .66 |
| Living alone, *n* (%) | 119 (66.11) | 33 (52.38) | *X*^2^(1) = 3.19; *p* = .07 |
| University, *n* (%) | 115 (64.24)^a^ | 36 (57.14) | *X*^2^(1) = 0.72; *p* = .40 |
| Paid work, n (%) | 116 (65.17)^b^ | 39 (62.90)^a^ | *X*^2^(1) = 0.03; *p* = .87 |
| **Clinical Variables** |  |  |  |
| Current psychological treatment (yes), *n* (%) | 61 (33.89) | 18 (28.57) | *X*^2^(1) = 0.38; *p* = .54 |
| Current medication (yes), *n* (%) | 32 (17.78) | 8 (12.70) | *X*^2^(1) = 0.55; *p* = .46 |
| Current psychiatric diagnosis, *n* (%) | 98 (54.44) | 36 (57.14) | *X*^2^(1) = 0.05; *p* = .82 |
| Depressive symptoms (PHQ-9), *M* (*SD*) | 8.74 (3.26) | 8.98 (3.45) | *t(241) = -0.49; p = .61* |
| Social Anxiety (SPS-6), *M* (*SD*) | 3.22 (3.62) | 3.58 (4.07)^a^ | *t(240) = -0.66; p = .51* |
| Social Interaction Anxiety (SIAS-6), *M* (*SD*) | 5.65 (4.13) | 6.21 (5.18)^a^ | *t(240) = -0.85; p = .40* |
| Personality Inventory (PID5BF+), *M* (*SD*) | 1.03 (0.29) | 1.09 (0.40)^a^ | *t(240) = -1.40; p = .16* |
| Childhood Trauma (CTQ)^f^ |  |  |  |
| Emotional abuse *n* (%) | 85 (47.22) | 29 (46.03) | *X*^2^(1) = 0.00; *p* = .99 |
| Physical abuse *n* (%) | 26 (14.44) | 13 (20.63) | *X*^2^(1) = 0.91; *p* = .34 |
| Sexual abuse n (%) | 38 (21.11) | 11 (17.74)^a^ | *X*^2^(1) = 0.15; p = .70 |
| Emotional neglect *n* (%) | 97 (53.89) | 37 (59.68)^a^ | *X*^2^(1) = 0.41; *p* = .52 |
| Physical neglect *n* (%) | 65 (36.11) | 28 (44.44) | *X*^2^(1) = 1.04; *p* = .31 |
| **Loneliness specific variables** |  |  |  |
| Loneliness at baseline (UCLA-9), *M* (*SD*) | 24.13 (3.20) | 24.84 (3.71) | *t(241) = -1.45; p = .15* |
| Duration of loneliness > 2 years, n (%) | 158 (89.77)^c^ | 54 (85.71) | *X*^2^(1) = 0.41; *p* = .52 |
| Suffering from loneliness | 2.26 (0.84)^a^ | 2.40 (0.99) | *t(240) = -1.04; p = .30* |
| Suffering from loneliness (dichotomized), n (%) | 148 (82.68)^a^ | 52 (82.54) | *X*^2^(1) = 0.00; *p* = 1.00 |
| Satisfaction with quantity of social relationships | 1.88 (0.77) | 1.84 (0.90) | *t(241) = 0.31; p = .76* |
| Satisfaction with quality of social relationships | 2.28 (0.82) | 2.06 (0.98) | *t(241) = 1.69; p = .09* |
| Social isolation (SNI) |  |  |  |
| Network size | 11.05 (6.06) | 11.17 (6.74) | *t(241) = -0.14; p = .89* |
| Network embeddedness | 1.58 (0.95) | 1.6 (1.09) | *t(241) = -0.18; p = .86* |
| Network diversity | 3.93 (1.50) | 3.86 (1.72) | *t(241) = 0.31; p = .76* |
|  |  |  |  |
|  | Completer  (n = 134) | Non-Completer  (n = 61) | Statistic |
| **Treatment related variables** |  |  |  |
| Modules accessed, *M* (*SD*) | 7.60 (2.02) | 3.82 (2.91) | *t(193) = 10.51; p < .001* |
| Time spent within the program (min), *M* (*SD*) | 585.66 (493.66) | 207.46 (226.81) | *t(193) =5.71; p < .001* |
| Working Alliance – Task&Goal (WAI), *M* (*SD*) | 3.19 (0.78)^d^ | 3.23 (0.63)^e^ | *t(157) = -0.24; p = .81* |

*Note.* GU = SOLUS-D with guidance; AM = SOLUS-D with automated message; WL = waitlist control group.

^a^ *n* of missings = 1

^b^ *n* of missings = 2

^c^ *n* of missings = 4

^d^ *n* of missings = 5

^e^ *n* of missings = 31

^f^ Participants with scores rated as “moderate to severe” or “severe to extreme” according to the assessment of severity of maltreatment (Häuser et al., 2011)

## References

Häuser, W., Schmutzer, G., Brähler, E., & Glaesmer, H. (2011). Maltreatment in childhood and adolescence: results from a survey of a representative sample of the German population. *Dtsch Arztebl Int*, *108*(17), 287-294. https://doi.org/10.3238/arztebl.2011.0287
